# Screening for distress, the 6th vital sign: common problems in cancer outpatients over one year in usual care: associations with marital status, sex, and age

**DOI:** 10.1186/1471-2407-12-441

**Published:** 2012-10-02

**Authors:** Janine Giese-Davis, Amy Waller, Linda E Carlson, Shannon Groff, Lihong Zhong, Eric Neri, Sacha M Bachor, Jassandre Adamyk-Simpson, Kate MS Rancourt, Bernie Dunlop, Barry D Bultz

**Affiliations:** 1 Department of Psychosocial Resources, Tom Baker Cancer Centre, Calgary, Canada; 2 Department of Oncology, University of Calgary, Calgary, California; 3 Department of Psychiatry and Behavioral Sciences, Stanford University, Halifax, Nova Scotia, Canada; 4 Department of Psychology, Dalhousie University, Stanford, California; 5 Department of Psychosocial Resources, Holy Cross Site, 2202 2nd St. S.W, Calgary, Alberta, T2S 3C1, Canada

**Keywords:** Marital status, Age, Sex, Cancer, Oncology, Screening for distress, Common problems

## Abstract

**Background:**

Very few studies examine the longitudinal prevalence of problems and the awareness or use of clinical programs by patients who report these problems. Of the studies that examine age, gender and marital status as predictors of a range of patient outcomes, none examines the interactions between these demographic variables. This study examined the typical trajectory of common practical and psychosocial problems endorsed over 12 months in a usual-care sample of cancer outpatients. Specifically, we examined whether marital status, sex, age, and their interactions predicted these trajectories. We did not actively triage or refer patients in this study in order to examine the natural course of problem reports.

**Methods:**

Patients completed baseline screening (*N* = 1196 of 1707 approached) and the sample included more men (*N* = 696) than women (*N* = 498), average age 61.1 years. The most common diagnoses were gastrointestinal (27.1%), prostate (19.2%), skin (11.1%) and gynecological (9.2%). Among other measures, patients completed a Common Problem Checklist and Psychosocial Resources Use questions at baseline, 3, 6, and 12 months using paper and pencil surveys.

**Results:**

Results indicated that patients reported psychosocial problems more often than practical and both decreased significantly over time. Younger single patients reported more practical problems than those in committed relationships. Younger patients and women of all ages reported more psychosocial problems. Among a number of interesting interactions, for practical problems, single older patients improved more; whereas among married people, younger patients improved more. For psychosocial problems we found that older female patients improved more than younger females, but among males, it was younger patients who improved more. Young single men and women reported the most past-and future-use of services.

**Conclusions:**

Younger women are particularly vulnerable to experiencing practical and psychosocial problems when diagnosed with cancer, but being married protects these younger women. Marriage appeared to buffer reports of both practical and psychosocial problems, and led to less awareness and use of services. Unexpectedly, young men reported the highest use of psychosocial services. This study informs clinical program development with information on these risk groups.

## Background

The National Comprehensive Cancer Network (NCCN) defines distress as an unpleasant psychological, social, and/or spiritual experience that interferes with effective coping [1]. Despite prevalence rates that can range from 35-60% in the cancer population [2-5], distress often goes unrecognized [6-10] and can have negative implications for patients including reduced health-related quality of life [11], greater long-term distress [12], poor satisfaction with medical care [13], and possibly reduced survival [14,15]. Common psychosocial, practical and physical problems may amplify feelings of distress, and their assessment offers clinicians opportunities to refer patients specifically to professionals who can address these patient problems. In this paper, we examine predictors of these common problems in order to facilitate strategic targets for offering psychosocial service.

Patients most frequently report physical, emotional and informational problems [11,16-18]; and these problems consistently predict clinical distress [3,11,17-19]. Some, but not all [18] researchers find that practical problems may also predict distress [3,11,19]. Other distressing problems may include social [20], financial [11,16,21], cognitive [3], sexual [22,23] and family related problems [3,11,16,24], and problems relating to the quality of cancer care received including care coordination [25] and relationships with health professionals [16,24,25].

Given the high prevalence of distress and common problems that may be present in the cancer population and the detrimental impact both can have on wellbeing, analyzing common problems and identifying characteristics of people who are more likely to report particular problems could facilitate planning for targeted clinical services [26]. Unmarried, younger and female patients report greater practical problems in mixed [21,24,27,28], as well as diagnostically homogenous populations [23,29,30]. Marriage also appears to buffer the number of problems patients report [27,31-34]. These associations include problems with finances [21,24,27,28], insurance [21,27,31], drug coverage [35] and employment issues [27,30,31]; as well as transportation [33], help around the home [27,33] and childcare [24]. Others report no differences in problems according to demographic characteristics [36].

Women report greater psychological, patient care and support [24,26,37], and sexuality problems [37]. Younger people report more psychological [24,27,31,37], patient care and support [38], information [24,27,31,37], social [21,27,31], physical and treatment-specific [21,31], sexual [23,30,37,38] and spiritual problems [24,27,31,37]. The association between marital status and psychosocial problems is less consistent; perhaps due to differing study settings and populations [16,29,39-41]. Variations in the measures used to assess problems, the population assessed, and the timing of assessments may contribute to these conflicting results [42].

Very few studies examine the longitudinal prevalence of problems and the awareness or use of clinical programs by patients who report these problems [26,38]. Of the studies that examine age, gender and marital status as predictors of a range of patient outcomes, none examines the interactions between these demographic variables. For example, do younger women experience psychological problems in the same way as older women and do these associations change over time? By examining how these demographic variables interact with each other, we may be better equipped to identify subgroups of people that may be more at risk for specific problems and facilitate the early identification and management of these issues before they become too overwhelming. As part of a comprehensive Screening for Distress the 6th Vital Sign in Cancer Care Program [43] adopted by a number of provinces across Canada, this study examined a naturalistic course of usual care without triage to document clinical outcomes and gaps in service. We have previously published usual-care baseline and longitudinal trajectories of distress, anxiety and depression, pain and fatigue [44], and this analysis not only adds to the literature, but also facilitates clinicians’ ability to directly modify the services they offer.

Because few studies examine common problems over time and their associations with distress, we first check these associations. We then test our primary hypotheses specifically examining associations between age, gender, and marital status as they interact and predict psychosocial and practical problems. Lastly, we examine secondary hypotheses relating to past, present, and future resource use.

### Check of associations between problems and distress

We examined whether practical and psychosocial problems correlated significantly with distress at baseline and over 12 months.

### Primary hypotheses

1. Being married, partnered, or in a committed relationship will buffer (or lower) reports of practical and psychosocial problems, both at baseline and over time.

2. Younger single, divorced, widowed, or separated women will represent a risk group for greater need in both practical and psychosocial problems.

### Secondary hypotheses

3. Due to these lower needs/problems, being married will lead to less awareness of and past, current, or anticipated use of psychosocial services. Due to these higher needs/problems younger or single women will report greater awareness, past, current, and anticipated use of services.

## Method

### Participants

**Figure 1:**
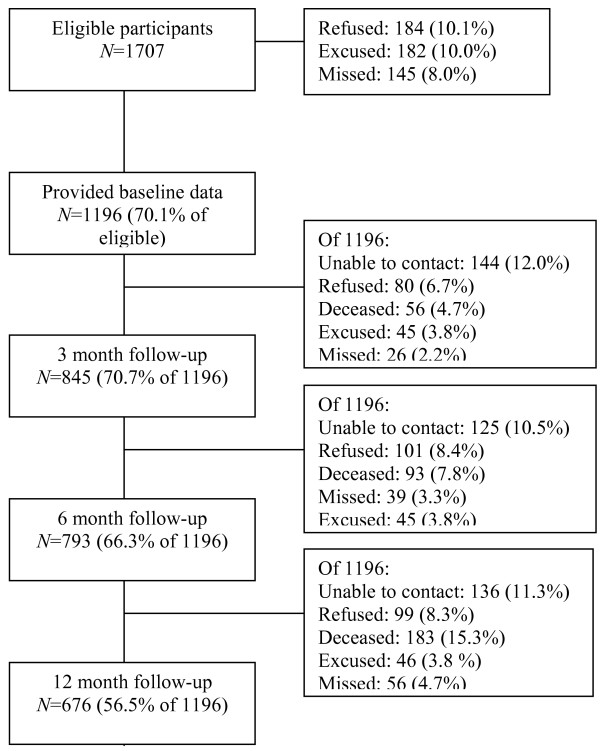
Study flow diagram.

Research assistants (RAs) approached ambulatory oncology patients (over 18) attending the Tom Baker Cancer Centre (TBCC) Outpatient Clinics who were new to TBCC, to that particular clinic, or to the scheduled oncologist, to participate in this study approved by the Conjoint Health Research Ethics Board of the University of Calgary. Research assistants excluded patients who did not read or speak English and did not have an interpreter with them, or patients deemed too ill (e.g., arrived in a stretcher). In total, 1196 (70%) patients signed informed consent and participated (511 of 1707 eligible were missed, excused, or refused to participate: Figure 1). A more detailed description of the study trial methodology has previously been reported [44,45].

### Measures

*Demographics and cancer history:* We assessed age, sex, marital status, living arrangements (alone or with others), education, ethnic/cultural background, income, source of income, first language, type of cancer and type of treatment, and the Alberta Cancer Registry provided information on whether patients had primary or metastatic diagnoses.

*The Modified Problem Checklist* (PCL). Adapted to the Canadian setting from the original list published by the NCCN, this list contains the 7 most common practical problems in our settings (accommodation, transportation, parking, drug coverage, work/school, income/finances, and groceries); and 13 psychosocial problems (burden to others, worry about family/friends, talking with family, talking with medical team, family conflict, changes in appearance; alcohol/drugs, smoking, coping, sexuality, spirituality, treatment decisions and sleep). Participants indicate the presence or absence of each problem in the preceding week [46].

*Awareness and Use of Psychosocial Resources.* Four questions assessed patients’ awareness and use of Psychosocial Resources: whether the patient is aware that a Psychosocial Department exists, whether the patient has used, or is currently using those services, and if the patient intends to use those services in the future.

*Distress Thermometer (DT):* Patients rated their average distress in the last week on a scale ranging from 0 “not at all” to 10 “extreme distress” [46]. The Distress Thermometer has been validated against the HADS, BSI, CES-D and clinical diagnosis in patients with mixed diagnoses and stages of disease [47]. A review of diagnostic validity studies reported a pooled sensitivity of 77.1% and specificity of 66.1% [48].

*The psychological screen for cancer (PSSCAN Part C*) [49,50]: Patients rated their anxiety and depression using 10 items rated on a 5 point Likert scale, ranging from “not at all” to “very much so”. [49,50]. Cronbach alphas ranged from .79 to .89 and test-retest stabilities ranged from .49 to .87 [49,50].

### Procedure

RAs assessed daily TBCC clinic lists and identified eligible patients. Once the patient checked in, they approached the patient to explain the study. If the patient consented to participate, the RA asked them to complete the questionnaires while at the clinic. Once completed, patients deposited the questionnaires into a designated box. If patients chose not to participate, they checked off their reason for not doing so (or the RA asked them and did this) and submitted the uncompleted questionnaires.

RAs contacted patients 3, 6, and 12-months later via e-mail or telephone. If the patient provided an e-mail address during their initial assessment, RAs sent them an automated email inviting them to complete the follow-up on-line. If they did not respond one week after the reminder, RAs added their name to the automatically generated phone list and contacted them by telephone. RAs made 3 calls at different times of the day, at least one of which was in the evening/weekend, before marking patients as “unable to contact”.

### Data analysis

First we examined the prevalence of common practical and psychosocial problems through baseline descriptive statistics. In order to examine baseline and over-time totals, we summed practical and psychosocial problems separately. We Winsorized these measures to adjust for a skewed distribution so that all summed total scores above 5 were set to equal 5 and examined baseline averages for each summary category.

#### Check of whether common problems correlate with distress

We examined the association between Practical and Psychosocial problems and patient reported distress, anxiety and depression at baseline using Spearman correlations. We then calculated a slope of outcome on time (measured in months) using standard linear regression [51] for each participant who provided data at baseline and at least one follow-up. We examined the association between Practical and Psychosocial problem slopes and each of the distress, anxiety and depression slopes using Spearman correlations (as effect-size estimates).

#### Primary analyses

We investigated associations between marital status, sex, and age and practical and psychosocial problems at baseline and over time using two Hierarchical Linear Model (HLM) equations with random-effect co-variance structures, one with practical problems as the dependent measure and one with psychosocial problems (Proc Mixed SAS). Independent variables included Time, Age (as a continuous variable), Sex, Marital Status (married/partnered vs. not married), and all interactions centered [52]. We report results for associations at baseline, and for interactions with time which represent change over time in dependent variables.

In secondary analyses, we examined whether Age, Sex, and Marital Status predicted Awareness, Past, Current, and Future Use of Psychosocial Resources. We conducted four logistic regressions with binary dependent variables representing the four Awareness and Use Categories. The independent variables were marital status, sex, age, and all the interactions, all centered [52].

Lastly, we examined whether stage of disease (primary vs. metastatic) and type of treatment (surgery, chemotherapy, radiation, hormone therapy) could explain our results. In order for our results to be proxies for these underlying prognostic variables, we would have to find significant correlations between the prognostic variables and both hypothesized independent (IVs) and dependent (DVs) variables. We tested these associations using Spearman Correlations. Any prognostic variable significantly correlated with both IVs and DVs would then be included in adjusted HLM and logistic regression models.

HLM models were analysed using SAS Version 9.2 (SAS Institute Inc., NC, USA, 2007). All remaining data were analysed using Statistical Package for the Social Sciences (SPSS) Version 19.

## Results

### Demographics and medical information

Of the 1707 patients we approached to participate during the recruitment period, 1196 (71%) provided baseline data (see Figure 1– recruitment diagram). The average age of the sample was 61.1 (*SD* = 14.5) years, 58% were male and 74% were married, common-law, or in a committed relationship (Table 1). The largest tumor groups were gastrointestinal (27.1%), prostate, (19.2%), and skin (11.1%). The majority of patients had primary (69%) rather than metastatic (13%) diagnoses.

**Table 1:** Participant demographic, medical, and study variables for participants in usual care study at baseline

**Demographic, medical, and study variables**	**Baseline (*n*** **= 1196)**	
	***N *or Mean**	**% or *SD***
**Mean number problems**		
Practical	0.94	1.37
Psychosocial	1.96	1.91
**Mean slope of change in problems**		
Practical	−0.05	0.18
Psychosocial	−0.03	0.25
**Psychosocial Resources (% endorsed)**		
Awareness	595	50.8
Past use	87	7.4
Current use	14	1.2
Future use	94	8.0
**Age (years)**	61.10	14.51
**Gender**		
Male	698	58.3
Female	498	41.7
**Marital status**		
Single	94	7.9
Married	779	65.1
Separated	32	2.7
Divorced	78	6.5
Widow/er	102	8.5
Common Law	66	5.5
Committed	22	1.8
Missing	23	1.9
**Living arrangements**		
Not Alone	969	84.1
Alone	183	15.9
**Education**		
Elementary School (1-6)	30	2.5
Middle School (7-9)	106	8.9
High School (10-12)	401	33.5
Community College	235	19.6
Some University	115	9.6
Completed University	167	14.0
Postgraduate	112	9.4
Missing	30	2.5
**Ethnicity**		
English as first language and visible majority	992	82.9
English as first language and visible minority	46	3.8
English not first language and visible majority	75	6.3
English not first language and visible minority	55	4.6
Missing	28	2.3
**Family income**		
Less than $30,000	215	18.0
Between $30,001 and $49,999	270	22.6
Between $50,000 and $79,999	173	14.5
Between $80,000 and $99,999	117	9.8
more than $100,000	180	15.1
Prefer not to say	195	16.3
Missing	46	3.8
**Source income**		
Pension/Retirement (CPP)	446	37.3
Employment	443	37.0
Family members (spouse/parent)	123	10.3
Social assistance	54	4.5
Prefer not to say	55	4.6
Other	38	3.2
Missing	37	3.1
**Diagnosis**		
Gastrointestinal	329	27.5
Prostate	230	19.2
Skin (melanoma)	133	11.1
Gynecological	110	9.2
Head and neck	75	6.3
Breast	69	5.8
Lymphoma	49	4.1
Leukemia	39	3.3
Testicular	33	2.8
Lung	31	2.6
Thyroid	25	2.1
Brain	25	2.1
Other	48	4.0
**Stage of disease**		
Primary	824	68.9
Metastatic	153	12.8
Missing/Not Staged	219	18.3
**Receipt of treatment prior to baseline**		
Surgery	275	23.0
Chemotherapy	100	8.4
Radiation therapy	32	2.7
Transplant	1	0.1
Hormone therapy	50	4.2

### Prevalence of common problems

**Figure 2:**
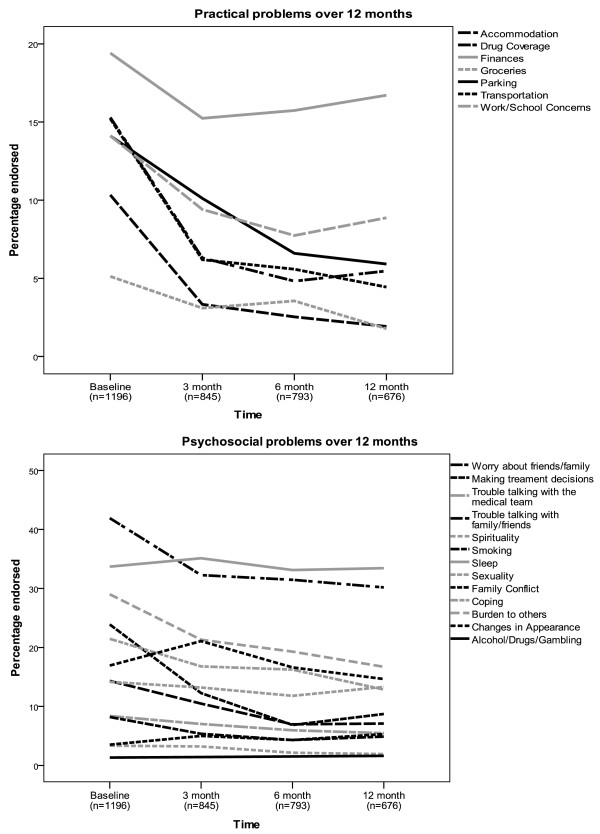
a and b: Percentage of patients endorsing Canadian problem Checklist items at each time point.

Figure 2a and 2b present the percentage of patients at each time point endorsing each of the problems on the CPC. Patients endorsed psychosocial problems at greater percentages (*M* = 16.9% for psychosocial; *M* = 13.4% for practical at baseline) and higher levels (*M* = 2.20, *SD* = 2.08) than practical (*M* = 0.94, *SD* = 1.37) problems. The top 4 highest percentages of endorsement were for psychosocial problems (Worry about friends/family (*M* = 42.0%), Sleep (*M* = 33.8%), Being a burden to others (*M* = 29.0%) and Coping (*M* = 21.4%)). The fifth highest was Finances (*M* = 19.4%), which were the most commonly endorsed of practical problems, and remained high over time (Figure 2a). Endorsement of Worry about friends/family and Sleep also remained high over time (Figure 2b).

#### Check of whether common problems correlate with distress

The number of practical problems reported at baseline was positively correlated with baseline distress, anxiety, and depression scores. The relationship between the number of psychosocial problems and distress , anxiety, and depression was stronger (Table 2).

**Table 2:** Correlations between total number of practical and psychosocial problems and distress thermometer (DT) scores at baseline and over time

	**Mean (*SD*)**	**Correlation with practical problems**	**Correlation with psychosocial problems**
**Baseline scores**			
Total Practical problems	0.94 (1.37)	.-	-
Total Psychosocial problems	2.20 (2.08)	.386**	-
Distress Thermometer (DT)	3.89 (2.89)	.278**	.526**
Depression (PSSCAN)	6.46 (3.07)	.225**	.444**
Anxiety (PSSCAN)	8.72 (4.09)	.270**	.508**
**Over 12 months (slope)**			
Total Practical problems	-.05 (0.29)	-	-
Total Psychosocial problems	-.05 (0.18)	.266**	-
Distress Thermometer (DT)	-.11 (0.47)	.114**	.356**
Depression (PSSCAN)	-.05 (0.34)	.098**	.221**
Anxiety (PSSCAN)	-.19 (0.52)	.124**	.324**

*p < 0.05; **p < 0.001.

Practical problems declined and correlated at a low level with distress, and anxiety over time, but the relationship with depression was very small (Table 2). Psychosocial problems slopes significantly correlated with distress, anxiety, and depression slopes with all outcomes declining over time (Table 2).

### Primary analyses

#### Baseline practical and psychosocial problems

**Figure 3:**
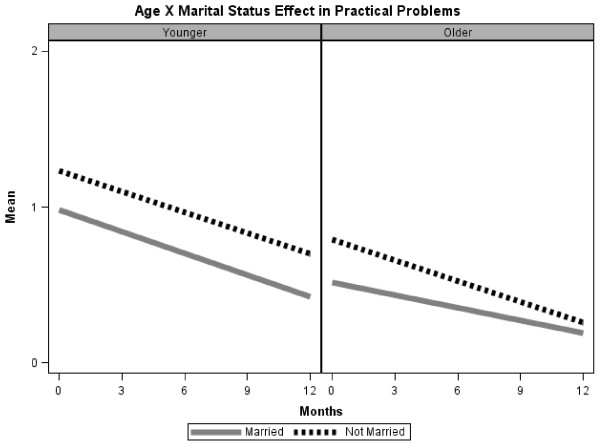
Two-way interaction (Age x Marital status) on changes in Practical problems over time.

**Figure 4:**
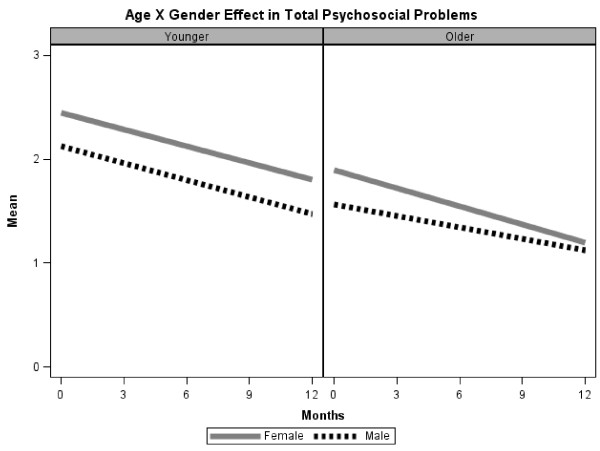
Two-way interaction (Age x Sex) on changes in Psychosocial problems over time.

In the HLM analysis, we found a significant 2-way interaction (Age x Marital Status) for practical problems (Table 3). Overall, younger patients reported more practical problems than older patients at baseline; within each age group, single patients reported more practical problems than married patients (Figure 3). We found no significant interaction in the HLM analysis of psychosocial problems at baseline. Main effects were that age and sex predicted psychosocial problems (Table 4); younger patients and females reported more psychosocial problems than males (Figure 4).

**Table 3:** Results from hierarchical linear model analysis of the impact of age, sex, and marital status on report of practical problems at baseline and over 12 months (N = 1196)

	**HLM on practical problems**			***ES***
	***Estimate***	***T-value***	***P***	
***Baseline***				
Age	−0.018	−8.08	<.0001	−0.02
Sex	−0.044	−0.60	0.55	−0.04
Marital Status	−0.299	−4.12	<.0001	−0.26
Age x Sex	−0.004	−0.92	0.36	−0.004
Age x Marital Status	−0.009	−2.05	0.04	−0.01
Sex x Marital Status	−0.248	−1.71	0.09	−0.22
Age x Sex x Marital Status	0.017	1.88	0.06	0.02
***Over 12 Months***				
Time	−0.04	−9.46	<.0001	−0.04
Age x Time	0.0005	1.70	0.09	0.0004
Sex x Time	0.0006	0.07	0.95	0.0005
Marital Status x Time	0.009	1.08	0.28	0.008
Age x Sex x Time	0.0001	0.25	0.80	0.0001
Age x Marital Status x Time	0.001	2.02	0.04	0.001
Sex x Marital Status x Time	0.021	1.26	0.21	0.02
Age x Sex x Marital Status x Time	−0.0006	−0.56	0.58	−0.001

**Table 4:** Results from hierarchical linear model analysis of the impact of age, sex, and marital status on report of psychosocial problems at baseline and over 12 months (N = 1196)

	**HLM on psychosocial problems**			***ES***
	***Estimate***	***T-value***	***P***	
***Baseline***				
Age	−0.02	−5.52	<.0001	−0.01
Sex	0.25	2.36	0.02	0.15
Marital Status	−0.01	−0.12	0.90	−0.01
Age x Sex	−0.0004	−0.06	0.95	−0.0002
Age x Marital Status	−0.01	−1.15	0.25	−0.01
Sex x Marital Status	0.33	1.58	0.11	0.20
Age x Sex x Marital Status	0.02	1.21	0.23	0.01
***Over 12 Months***				
Time	−0.05	−7.27	<.0001	−0.03
Age x Time	0.0002	0.49	0.62	0.0001
Sex x Time	−0.007	−0.56	0.57	−0.004
Marital Status x Time	−0.03	−2.05	0.04	−0.02
Age x Sex x Time	−0.002	−2.09	0.04	−0.001
Age x Marital Status x Time	−0.001	−0.63	0.53	−0.001
Sex x Marital Status x Time	−0.03	−1.38	0.17	−0.02
Age x Sex x Marital Status x Time	0.001	0.56	0.58	0.001

#### Change over 12 months in practical and psychosocial problems

In HLM analyses (Table 3), a main effect of time indicated that practical problems decreased significantly during the 12 months of the study. A significant 3-way interaction (Age x Marital Status x Time) indicated that for singles, older patients improved more than younger patients over time; whereas for married people, younger patients improved more over time). Younger, single patients were the most elevated at baseline and remained so at 12 months (Figure 3).

In HLM analyses (Table 4), a main effect of time indicated that psychosocial problems decreased significantly during the 12 months of the study. A significant 3-way interaction (Age x Sex x Time) for psychosocial problems indicated that among females, older patients improved more over time in psychosocial problems; among males, younger patients improved more over time (Figure 4). Younger females were the most elevated at baseline and remained the most elevated at 12 months. Main effects were that married patients declined more than single patients (Table 4).

### Secondary analysis

#### Awareness, past, current, and future use of psychosocial resources

Awareness of Psychosocial Resources available through Cancer Care at baseline was significantly related to sex; more females (53.1%) than males (49.1%) reported awareness (*OR* = 1.328, *SE* = 0141, 95% CI (1.008, 1.750), *p* = .043). Current use of Psychosocial Resources available through Cancer Care was not significantly related to Age, Sex, and Marital Status (though a trend for marital status for current use would suggest that fewer married/partnered men and women were using Psychosocial Resources (*OR* = 0.32, *SE* = 0.603, 95% CI (0.099, 1.050), *p* = .06).

More single (11%) than married people (6.2%) reported past use of Psychosocial Resources (*OR* = 0.532, *SE* = 0.254, 95% CI (0.323, 0.876), *p* = .013). The three-way Age x Sex x Marital Status interaction suggests that more young single men (18.8%) than young single women (10.3%) had used Psychosocial Resources, whereas the reverse was true in older participants (female: 9.3%; males: 4.6%). Moreover, younger married males (3.5%) reported using Psychosocial Resources less than younger married females (10.6%) (*OR* = 0.937, *SE* = 0.031, 95% CI (0.882, 0.996), *p* = .035). Lastly, more young (33.5%) (*OR* = 0.963, *SE* = 0.009, 95% CI (0.946, 0.980), *p* < .001) and single (37.3%) (*OR* = 0.36, *SE* = 0.277, 95% CI (0.186, 0.551), *p* < .001) men and women reported that they would use Psychosocial Resources in the future compared to older (14.8%) and married (18.2%) men and women.

### Are age, gender, and marital status proxies for prognostic variables?

Stage and Treatment did not simultaneously correlate significantly with IVs and DVs for any of our analyses. Because this condition was not met, and they could not be considered proxies, we did not adjust our HLM analyses.

## Discussion

We found that marital status buffers or reduces common problems, as it often buffers cancer patients’ distress [3,40] and unmet needs [27,31-34]; however, age differences impacted this relationship. Similarly, older age buffers common practical problems but differences exist between males and females. Lastly, not only younger women, but also younger men reported higher past and future use of our Psychosocial Resources Program. Stage of disease and type of treatment did not explain our results.

Analyzing common problems over time and identifying people at risk for common problems may guide clinicians in targeting interventions toward people who need them most [26]. It allows us to deconstruct elements of need so that we can offer appropriate practical as well as psychological help. For instance, in this study our findings suggest that providing younger single males and females access to practical support (e.g. help with finances), and younger men but women of all ages greater access to psychological support may be beneficial. These associations and interventions based on these findings may also impact distress as we found that the more psychosocial and practical problems patients reported, the higher their distress, anxiety, and depression levels at baseline. Psychosocial and practical problems declined over time and correlated with declines in distress, depression and anxiety.

This study is the first longitudinal investigation of common problems in patients new to the TBCC. The large sample size and longer follow-up period have enabled us to refine previous knowledge in this area. Similar to others, we found that common practical problems include finances and drug coverage [21,27,29,35], with work/school only a concern for younger people [27]. At baseline older people had considerably fewer problems, as did married people, resulting in the highest prevalence of practical problems in young, single people, particularly women. Marital status findings are consistent with other reports in the literature [21,27,33,53], but we add interactions with age to extend this literature. Younger and single people may have lower incomes, less financial stability if they need to leave work for long periods of time, and greater responsibility for young children. Cancer may disrupt their ability to handle all of these competing demands, so practical help might lead to the most positive improvements.

For psychosocial problems, younger patients reported greater psychosocial problems than older patients, while women reported greater psychosocial problems than men at baseline. Few psychosocial resources target specifically young men and women with support groups or counseling interventions [54,55], and anecdotally young patients often report that they feel they have nothing in common with older people with cancer in support groups. Again, other reports have consistently shown more psychosocial problems in women [24,26,37], perhaps due to an under-reporting bias in males [34,56] or due to a greater tendency in women to focus on processing emotions [57].

The picture becomes more complex when we look at our novel data investigating changes over 12 months. In general, practical and psychosocial problems improved, with the sharpest improvement in the first three-months, although some problems did not ease on their own. Patients endorsed financial problems often and endorsements remained high over time, even though in Canada patients have public health benefits. Younger, single patients endorsed the most practical problems at baseline and remained the most elevated at 12 months. Younger females endorsed the most psychosocial problems at baseline and remained the most elevated at 12 months. Perhaps these results indicate that neither group currently receives the help they need. Interventions that include help with practical aspects of going through treatment for young single women might improve their distress.

Of the psychosocial problems, worry about friends and family, and difficulty with sleep, remained high as patients underwent a variety of treatments. It is interesting to note that the most common psychosocial problem endorsed was worry about friends and family, a difficulty rarely addressed by health practitioners. Endorsement of this worry also did not decline dramatically over time, with over 30% still reporting it at 12 months. Older males improved the least in psychosocial problems; however, they did not report high levels of problems at baseline.

Lastly, report of use of the Psychosocial Resources Department at baseline reflected some of these findings. More young single men than young single women reported using Psychosocial Resources, whereas the reverse was true in older participants. This is an unusual finding, perhaps reflecting the prominent erectile dysfunction services offered, and further investigation could document which services young men accessed throughout the year. More young and single men and women reported interest in future use. These results mimic their reports of greater problems. However, older patients may not access services due to experiencing greater barriers in transportation or low caregiver help [58]. Apparently, being married reduces rates of reported current and future interest in the use of services, reflecting some of the buffering seen in the low endorsements of practical problems in married people. Future research could also examine the tumour type of the younger people endorsing use of psychosocial resources which is beyond the scope of this paper. Additionally, future research could examine whether older patients perceived greater barriers to access.

These new findings have implications for cancer-care teams who may want to prioritize resources at the time of initial diagnosis toward helping young, single men and women access services to help with resource and financial concerns. For younger single patients, a diagnosis of cancer could result in significant loss in income while expenses such as child care, food, and transportation continue to accrue. These findings challenge us to examine whether resources provided are sufficient for the burden these groups experience. Linking with community programs, providing appropriate childcare or housekeeping services, and enhancing support for basic needs could help significantly reduce the burden and distress of these at-risk groups. Older single patients improved to a similar level as older married patients in this study--they may be more able to handle practical concerns as they suffer less income loss if retired, tend to be more financially stable, and have good medical coverage here in Canada. However, examining carefully barriers to access is important to consider when providing services to older people.

Women of all ages need psychosocial support at the time of diagnosis, but we should not neglect younger men, as they have high practical needs at diagnosis and are using our Psychosocial Resources even more than young women. Innovative psychosocial programs could target this group that is at-risk for sustained problems, since historically men are less likely to access supportive care services on their own. Our care team offers specific therapy programs for men with prostate cancer who have erectile dysfunction which may, in part, account for this unusual finding [59,60]. Discussion groups or educational opportunities, that provide a point of entry into the care system, may also be attractive to men. When administrators better understand the needs of a range of patient groups, they can develop more suitable and effective programs. Clinically, these data identify problems and risk groups so intervention can happen earlier--which may result in improved coping and savings to the health care system as fewer crisis interventions may occur.

Although this study has strengths, including a large sample size, relatively high accrual rate, varied cancer diagnoses, and 12-month follow-up, only 72% of eligible participants consented to the study. There was a significant drop-out or missing data rate, which resulted in only 56.5% of the original sample (676/1196) being assessed at the final follow-up: some lost due to death or progression of illness, others missed follow-ups, a small group did not continue. However, HLM analysis mitigated this loss in generalizability by using data from all patients who provided at least one assessment in the analysis. The sample is also not representative of breast and lung patients because these tumour groups attended an outpatient clinic in a different location. This study used a measure that assessed the presence of problems in the week prior to questionnaire completion. Jacobsen et al. (2005) suggests that assessing those problems for which individuals want assistance may be more beneficial [18].

Lastly, because this is a mixed cancer sample in a usual-care cancer centre setting, the frequency of cancer types varied. Our goals were to provide evidence for a general cancer population and services offered in a general cancer setting. Certain cancers (testicular, prostate, breast) are gender-specific. It is not possible to adjust for this in an analysis examining gender. However, a larger number of participants in the current study had cancers that are not gender-specific (747 of 1196). Some cancers are age-correlated, and again, it is not possible to adjust for this in an analysis examining age. Other cancers are too rare in this sample for adjustment. As such, age effects may in part reflect the influence of those types of cancers that are age-dependent, and gender effects may in part reflect the influence of those types of cancers that are gender-specific. Further research could investigate larger samples of non-gender- and age-specific cancers.

## Conclusions

As part of a comprehensive Screening for Distress program, we investigated these associations between common practical and psychosocial problems and interactions with marital status, sex, and age during usual care. Although patients completed a screening questionnaire, we did not triage with referral to psychosocial resources, as our goals were to provide a naturalistic picture. This work informs clinical programming decisions, and we have identified several risk groups (young single women, and young men) to target with innovative interventions.

## Competing interest

The authors declare that they have no competing interests.

## Authors' contributions

Study conception and design: LEC, SLG, BDB, JGD. Provision of study materials or patients: LEC, SLG, BD. Collection and assembly of data: SMB, JAS. Data analysis and interpretation: JGD, AW, LZ, EN. Manuscript writing: JGD, AW, KR. All authors read and approved the final manuscript.

## Pre-publication history

The pre-publication history for this paper can be accessed here:

http://www.biomedcentral.com/1471-2407/12/441/prepub
